# Toddler Screen Time: Longitudinal Associations with Autism and ADHD Symptoms and Developmental Outcomes

**DOI:** 10.1007/s10578-024-01785-0

**Published:** 2024-11-29

**Authors:** Monique Moore Hill, Devon N. Gangi, Meghan Miller

**Affiliations:** Department of Psychiatry & Behavioral Sciences, UC Davis Medical Center, MIND Institute, University of California, 2825 50th Street, Sacramento, CA 95817 USA

**Keywords:** Attention-deficit/hyperactivity disorder (ADHD), Autism spectrum disorder (ASD), Neurodevelopment, Preschool, Screen Media

## Abstract

Greater screen time is associated with increased symptoms of autism spectrum disorder (autism), attention-deficit/hyperactivity disorder (ADHD), and lower scores on measures of development in preschool-aged community samples. In the current longitudinal study, we examined screen time differences at 18 months of age based on clinically-defined outcomes (i.e., Autism, ADHD Concerns, Comparison) determined at age 3–5 years in a genetically-enriched sample based on family history, along with prospective associations between toddler screen time and preschool autism/ADHD symptoms and developmental achievement. Participants (*n* = 82) included children at high and low familial likelihood for autism and ADHD. Children with Autism and ADHD Concerns outcomes experienced significantly more screen exposure at 18 months than children without autism or elevated symptoms of ADHD. Greater screen time at 18 months was also associated with preschool symptoms of autism and ADHD and lower developmental achievement across the sample. Preschoolers with neurodevelopmental challenges experienced more screen exposure earlier in development than same-age peers, increasing potential for negative developmental impacts.

## Introduction

Use of digital media among toddlers is prevalent worldwide [33]. Screen media is a routine component of family life, but evidence suggests early screen time may negatively impact development [47], creating a tension for parents. Although the American Academy of Pediatrics (AAP) and World Health Organization (WHO) recommend avoiding screen use prior to age 2 [12, 64], 75% of children under age 2 exceed daily recommendations [33] and less than half of mothers in the U.S. demonstrate a clear understanding of the guidelines [30].

Associations between screen time and early developmental outcomes may be particularly relevant for children experiencing neurodevelopmental challenges related to autism spectrum disorder (autism) or attention-deficit/hyperactivity disorder (ADHD), childhood conditions that co-occur with high frequency and share heritability [22, 37]. Increased screen time, together with the challenges inherent to autism and ADHD, may cumulatively impact developmental outcomes, and ensuring that parents are aware of recommendations to avoid screen time early in development may help support rather than further inhibit emerging skills in children showing neurodevelopmental differences. Engagement with digital media reduces opportunities for social interactions [4, 8, 45, 62] that promote optimal toddler development [24] and may amplify social and behavioral challenges [58] and negatively impact the development of self-regulation skills [9]. Studies in preschool-aged community samples demonstrate a longitudinal dose–response relationship between screen time and developmental outcomes [34, 56, 57], with greater screen time associated with lower parent-reported developmental achievement [32, 65]. However, neurodevelopmental differences were not assessed in these samples. Other longitudinal community-based studies show associations between toddler screen time and both autism [1, 18, 29] and ADHD symptoms [7, 58], also based on parent report. However, these studies did not directly assess developmental outcomes, and the directionality of any effects of screen time on autism and ADHD symptomatology remains unclear [40, 59]. To our knowledge no existing longitudinal studies in this age group examine both developmental achievement and neurodevelopmental symptoms, incorporating clinical assessment as part of the study design.

The present longitudinal study examines screen time in a sample of toddlers at increased likelihood for developing autism or ADHD based on family history, using a prospective familial likelihood design, including direct clinical assessment. These designs capitalize on the increased recurrence rates of autism and ADHD within families [37, 42], focusing on infants who have a diagnosed first-degree relative. These samples are enriched with a range of symptom presentations in early development, making this design an efficient strategy for identifying a broad range of symptom presentation early in development. Indeed, such studies have greatly contributed to the current understanding of autism [55] and ADHD [38] symptom presentation prior to age 3. One previous study examined screen time in a similar population, demonstrating cross-sectional associations between screen time and both ADHD symptoms and language development at age 3 [21].

The present study has two aims: (1) to assess differences in mean screen time at 18 months of age based on directly-assessed preschool clinical outcome classifications of Autism, ADHD Concerns, and a Comparison group; and (2) to examine longitudinal associations between screen time at 18 months and continuous measures of autism symptoms, ADHD symptoms, and developmental achievement in the preschool period.

## Method

### Overview of Procedures

This study utilized data from a longitudinal investigation of children at increased familial likelihood for autism and ADHD. Data was collected between 2017 and 2022. The University Institutional Review Board approved the research and parents provided written informed consent. Parents received monetary compensation for their time. All infants were enrolled by 18 months of age (mean age of enrollment = 13.74 months, SD = 2.76). Developmental assessments occurred at 12, 18, 24, and 36–64 months of age. Between 2020 and 2022, COVID-19 social distancing mandates necessitated modification of the original study design including: (1) extending the window for the final diagnostic assessment from the originally intended age of 36 months to age 3–5 years (69.9% were completed prior to 48 months of age, and 98% were completed prior to 60 months of age), and (2) using an alternate validated measure for autism diagnosis for a portion of the sample (described in the measures section below). Masters- or Ph.D.-level examiners with demonstrated reliability of 80% or greater on all measures conducted assessments with clinical oversight from a licensed clinical psychologist regarding clinical outcome determination and without awareness of familial risk group, previous test results, or diagnoses. The current analyses utilized data from the 18-month and 3–5-year assessments.

### Participants

Families were recruited from the community in the greater Sacramento region. The sample included children at higher likelihood to develop autism or ADHD based on the diagnostic status of an older sibling (autism and ADHD likelihood groups) or parent (ADHD likelihood group only), as well as children at low likelihood for either condition based on family history considering first-, second-, and third-degree relatives.

Data analyzed for the purposes of this study were collected prospectively at 18 months of age (M = 17.83, SD = 0.33) and 3–5 years of age (M = 42.79 months, SD = 8.29). All 18-month data and 37.8% of 3–5-year data were collected prior to the COVID-19 pandemic. Participants were classified into one of three mutually exclusive analysis groups based on a standardized clinical outcome evaluation at age 3–5. The Autism outcome group met *Diagnostic and Statistical Manual of Mental Disorders, 5th Edition* (*DSM-5*; [2]) criteria for autism spectrum disorder and obtained a score over the autism spectrum disorder cutoff on the Autism Diagnostic Observation Schedule, Second Edition (ADOS-2; [31]) or, when necessary due to masking requirements during the COVID-19 pandemic, the Brief Observation of Symptoms of Autism (BOSA; [13]). Following the approach of previous studies [17, 21, 48], the ADHD Concerns group exhibited ≥ 4 *DSM-5* symptoms of either inattention or hyperactivity, or ≥ 5 symptoms across domains with at least one symptom endorsed by multiple raters (i.e., parent, teacher, examiner), and a clinical best estimate outcome of “ADHD Concerns” based on the diagnostic assessment. Symptoms of ADHD begin to emerge early in development, indicating elevated risk for later diagnosis [36], however, given the young age of our sample, most did not yet meet full *DSM-5* ADHD diagnostic criteria. Children with elevated ADHD symptoms who also had an autism diagnosis (*n* = 6) were included in the Autism group based on their primary diagnosis of autism. The Comparison group included all other participants who did not meet criteria for Autism or ADHD Concerns.

The final analyzed sample included a total of 82 participants: *n* = 15 with Autism outcomes, *n* = 17 with ADHD Concerns outcomes, and *n* = 50 with Comparison outcomes. Sample characteristics by outcome group are presented in Table 1.

**Table 1:** Sample characteristics by outcome group

	Autism	ADHD concerns	Comparison
*Recruitment Group*	*n* (%)	*n* (%)	*n* (%)
Familial ASD	15 (100%)	2 (12%)	15 (30%)
Familial ADHD	0 (0%)	10 (59%)	11 (22%)
No Familial ASD/ADHD	0 (0%)	5 (29%)	24 (48%)
*Sex*			
Male	9 (60%)	8 (47%)	30 (60%)
*Ethnicity*^*a*^			
Hispanic/Latino	1 (7%)	2 (12%)	6 (12%)
Non-Hispanic/Latino	13 (87%)	15 (88%)	42 (84%)
*Race*^*b*^			
American Indian or Alaskan Native	0 (0%)	0 (0%)	1 (2%)
Asian	1 (7%)	0 (0%)	2 (4%)
More than one race	5 (33%)	3 (18%)	12 (24%)
White	9 (60%)	14 (82%)	31 (62%)
*Maternal Education*^*c*^			
No college degree	7 (47%)	2 (12%)	6 (12%)
College degree or higher	8 (53%)	14 (82%)	44 (88%)
*Income*^*d*^			
≤ $80,000	4 (27%)	4 (24%)	14 (28%)
> $80,000	10 (67%)	10 (59%)	34 (68%)
	M (SD)	M (SD)	M (SD)
*Total Screen Time (hours), 18 months*	1.65 (1.07)	1.21 (0.97)	0.68 (0.75)
*Exceeded AAP Guidelines, 18 months*	15 (100%)	16 (94%)	36 (72%)
*MSEL Subscale T-scores, 3–5 years*^*e*^			
Visual Reception	43.27 (14.20)	52.94 (10.23)	57.75 (10.54)
Fine Motor	37.33 (14.27)	46.94 (11.31)	57.15 (14.78)
Receptive Language	34.67 (11.00)	46.76 (7.72)	52.33 (9.72)
Expressive Language	38.40 (12.37)	47.82 (10.42)	52.44 (10.13)
*SRS Total Score, 3–5 years*^*f*^	59.53 (15.00)	50.53 (8.84)	45.23 (5.59)
*Preschool ADHD Rating Scale Total Score, 3–5 years*^*g*^	20.07 (12.81)	24.71 (8.95)	8.30 (6.52)

*AAP* American Academy of Pediatrics; *MSEL* Mullen Scales of Early Learning; *SD* standard deviation; *SRS* Social Responsiveness Scale

Missing data for: ^a^1 participant in the Autism group and 2 in the Comparison group

^b^4 in the Comparison group

^c^1 in the ADHD Concerns group

^d^1 in the Autism group, 3 in the ADHD Concerns group, and 2 in the Comparison group

^e^2 in the Comparison group

^f^7 in the Comparison group

^g^4 in the Comparison group

### Measures

#### The Attention-Deficit/Hyperactivity Disorder Rating Scale–Preschool Version (ADHD-RS; [35])

The ADHD-RS, Preschool Version measures symptoms of hyperactivity and inattention in children ages 3–5 with high internal consistency (Cronbach’s alpha for parent version = 0.92, teacher version = 0.95) and test–retest reliability (parent version = 0.87, teacher version = 0.94). This questionnaire was completed at the outcome visit by parents and, when possible, an additional rater (e.g., daycare provider, preschool teacher) to determine diagnostic group classification using symptoms counts. The total parent rating score was also analyzed as a continuous measure of ADHD symptomatology.

#### Autism Diagnostic Observation Scale, Second Edition (ADOS-2; [31])

The ADOS-2 is a widely used semi-structured play-based interaction and observation with high inter-rater reliability and agreement in diagnostic autism classification (autism spectrum vs. non-spectrum). The ADOS-2 was administered to determine outcome classification (*n* = 31).

#### Brief Observation of Symptoms of Autism (BOSA; [13])

The BOSA is a standardized, semi-structured set of play activities administered by a caregiver or clinician and scored by an ADOS-trained clinician to determine diagnostic autism classification (autism spectrum vs. non-spectrum). BOSA scores demonstrate high inter-rater reliability (intraclass correlation coefficients [ICC] = 0.90–0.94), test–retest reliability (ICC = 0.95), and convergent validity with the ADOS-2 (correlation of 0.74). Parents and children completed the BOSA in the lab while being observed by an ADOS-reliable examiner from behind a one-way mirror. Scores were used to determine outcome classification in place of the ADOS-2 when masking guidelines during the COVID-19 pandemic prevented administration of the ADOS-2 (*n* = 51).

#### Mullen Scales of Early Learning (MSEL; [39])

This standardized developmental test for children birth to 68 months was administered to all participants. MSEL subscales have excellent internal consistency (median 0.91) and test–retest reliability (median 0.84). The Visual Reception, Fine Motor, Receptive Language, and Expressive Language subscales were administered, and subscale *T*-scores were used for analyses.

#### Screen Time

Parents indicated the number of hours their child spent watching television programs, movies, and streaming media content on any device on a typical weekday and weekend day (rating scale: 0, < 1, 1, 2, 3, or >  = 4 h per day). Responses of < 1 h and ≥ 4 h were coded as 0.5 and 4 respectively. Following prior work [32, 67], we calculated a weighted daily average by multiplying the weekday response by 5, the weekend response by 2, and dividing the sum of these by 7, resulting in a possible range of 0 to 4 h per day.

#### Social Responsiveness Scale, Second Edition (SRS-2; [10])

The SRS-2 is a widely used parent report questionnaire that measures social skills and behavior associated with autism in children 2–18 years of age. The SRS-2 has excellent internal consistency (0.94) and interrater agreement (0.77). It was administered when participants were 3–5 years old. Total scores were analyzed as a continuous measure of autism symptomatology.

### Analytic Plan

To examine differences in screen time hours between outcome groups, analysis of variance (ANOVA) was employed, followed by planned comparisons between groups. To control for potential effects of relevant demographic factors on screen time [14], sex, maternal education, income, and race (white/non-white)^1^ were dummy-coded and added as covariates to the models; however, only maternal education was significant and therefore retained in the final ANOVA model. To then examine longitudinal associations between screen time at 18 months and preschool autism/ADHD symptoms and developmental outcomes, regression analyses were employed. Potential covariates (sex, maternal education, income, and race [white/non-white]) were dummy-coded and tested in the models; none were significant and therefore were not retained in final models.

## Results

**Fig. 1:**
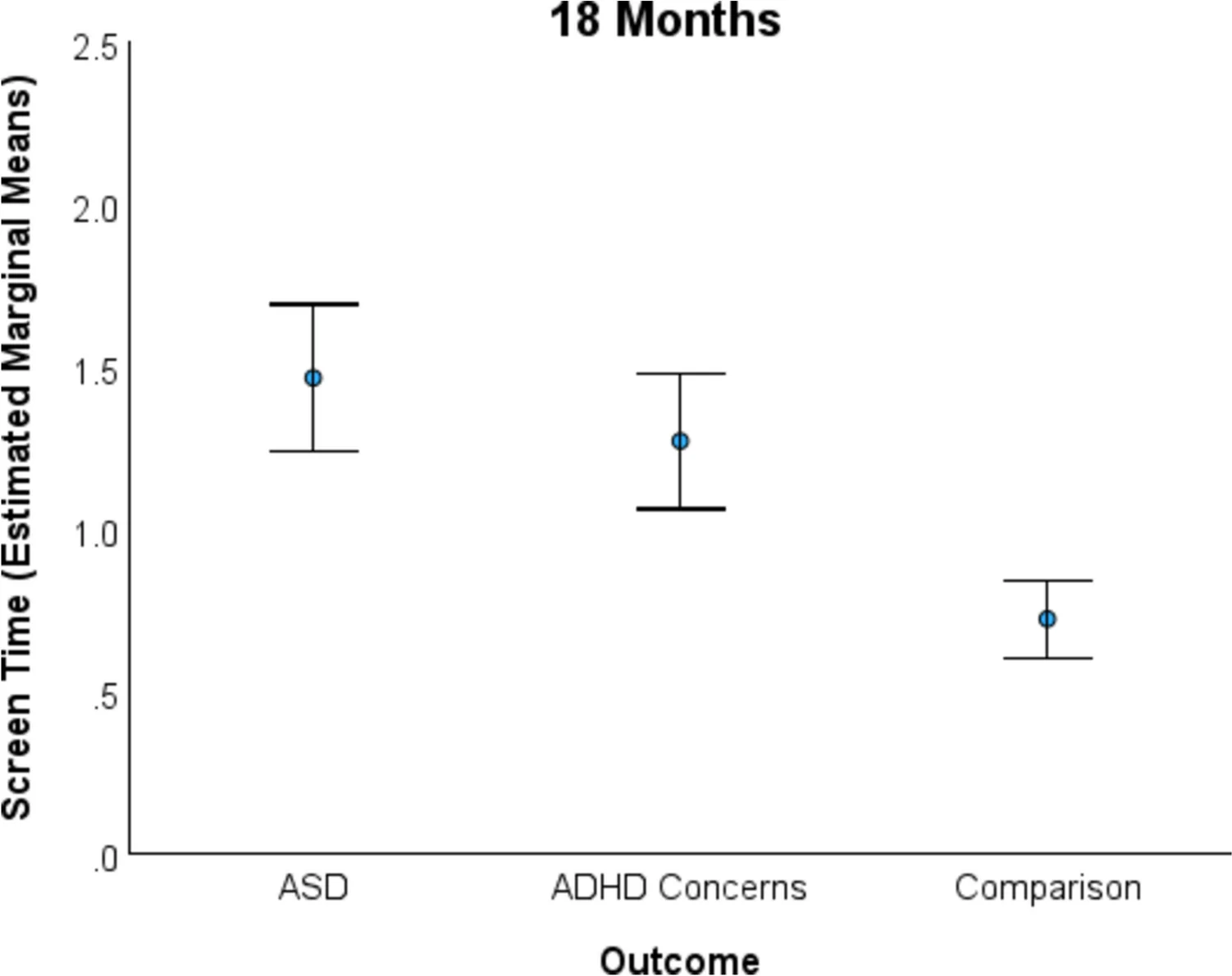
Estimated marginal means of screen time by outcome group. *Note* Error bars represent ± 1 standard error

We first examined group differences in total screen time hours at 18 months of age by preschool outcome group, finding a significant effect of outcome group on screen time after adjusting for maternal education, *F*(2, 77) = 5.45, *p* = 0.006, partial η^2^ = 0.12 (see Fig. 1). Post-hoc analyses indicated this main effect was a result of significantly greater screen time hours in the Autism group (adjusted *M* = 1.47, Standard Error (*SE*) = 0.23) relative to the Comparison group (adjusted *M* = 0.72, *SE* = 0.12), *p* = 0.006, and in the ADHD Concerns group (adjusted *M* = 1.27, *SE* = 0.21) relative to the Comparison group, *p* = 0.025. The ADHD Concerns and Autism groups did not differ, *p* = 0.536.

**Fig. 2:**
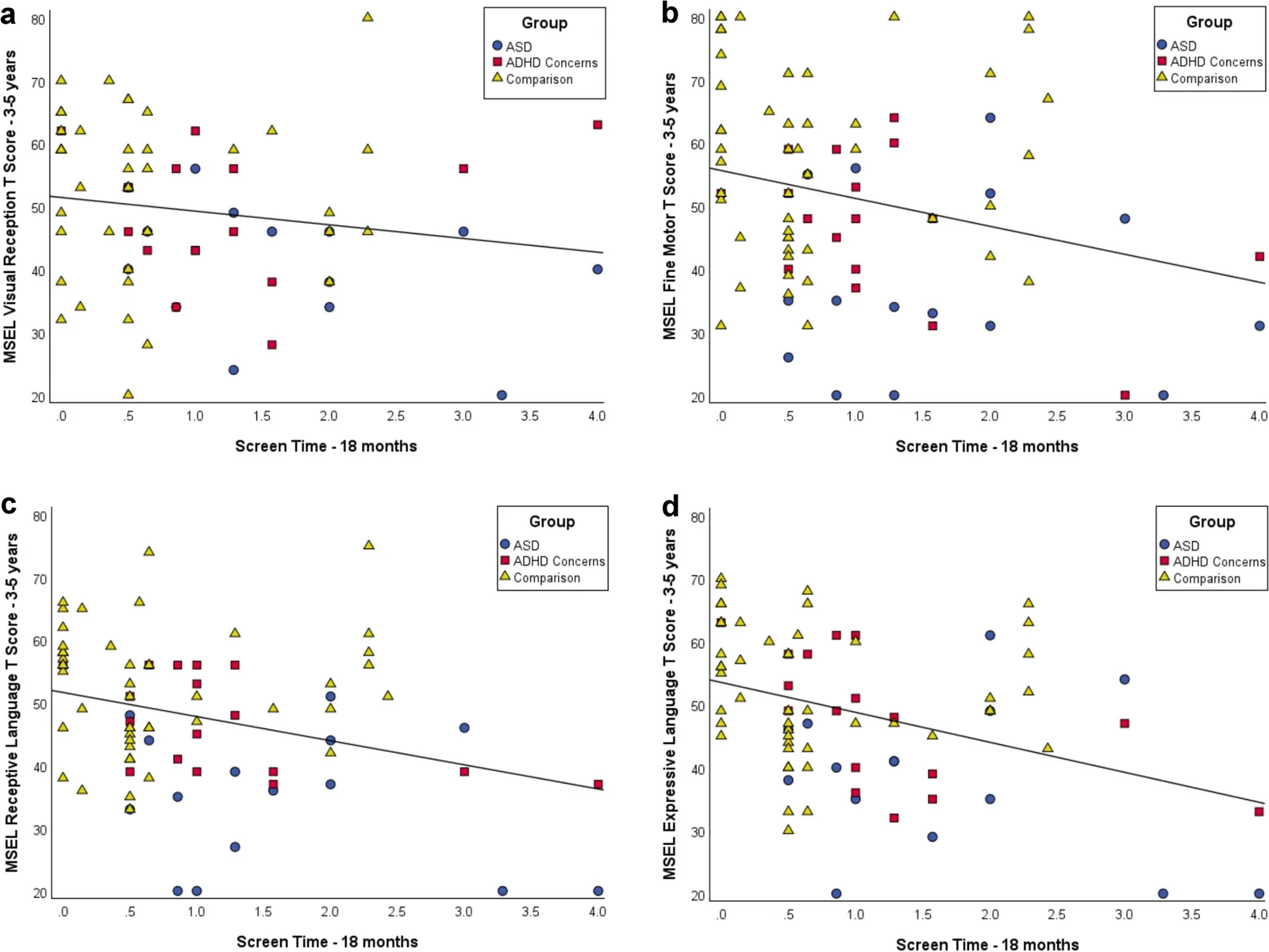
Scatterplots of associations between screen time at 18 months and MSEL scores at 3–5 years: **a** Visual Reception, **b** Fine Motor, **c** Receptive Language, and **d** Expressive Language

**Fig. 3:**
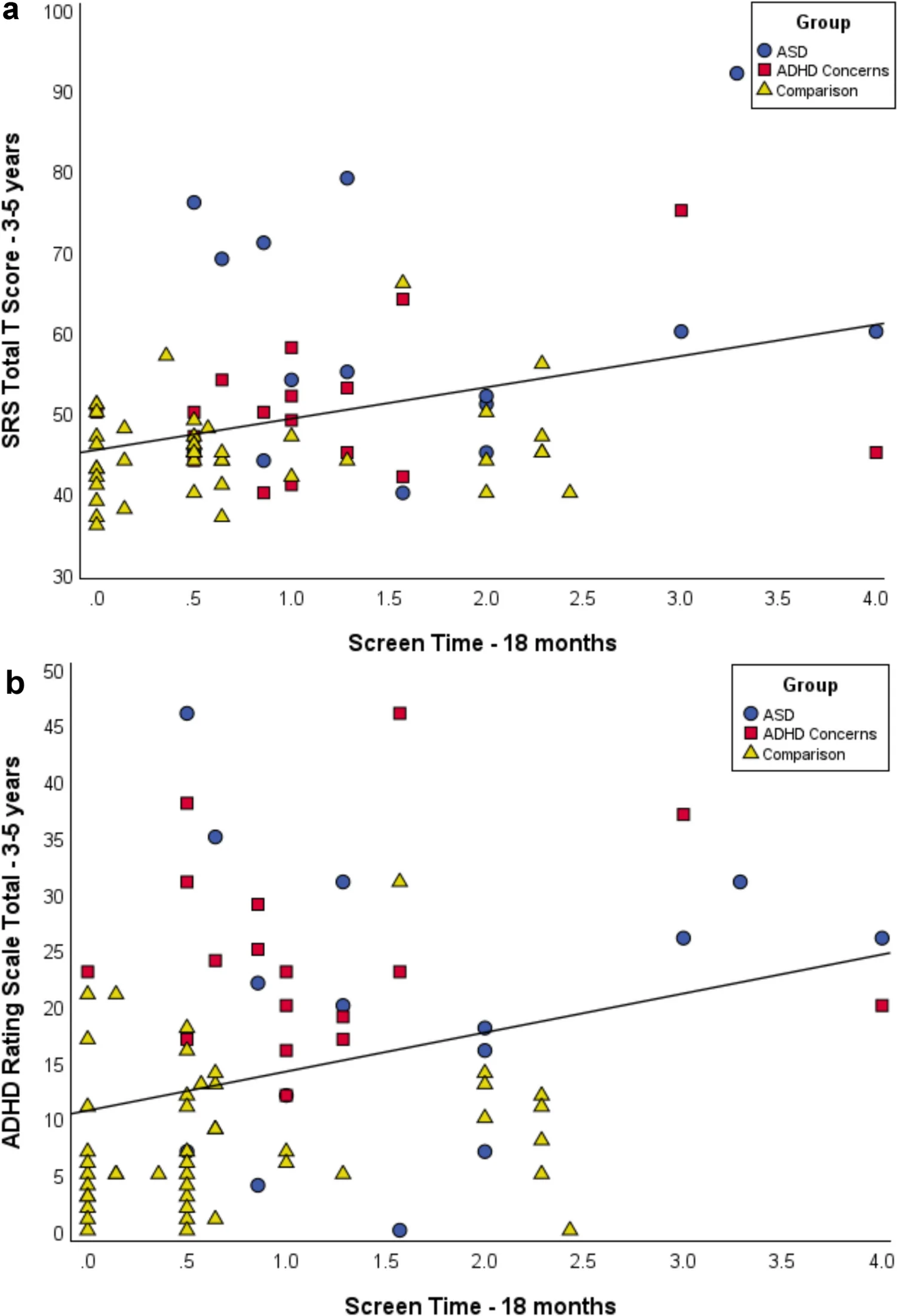
Scatterplots of associations between screen time at 18 months and **a** autism symptoms and **b** ADHD symptoms at 3–5 years

Next, we examined associations between 18-month screen time, developmental scores and preschool autism/ADHD symptoms. Regression coefficients for the analyses predicting continuous scores at 3–5 years from screen time at 18 months are presented in Table 2 and scatterplots are presented in Figs. 2 and 3. Screen time was significantly associated with all Mullen subscale scores: Visual Reception, β =  − 0.23, *SE* = 1.46, *p* = 0.044, Fine Motor, β =  − 0.26, *SE* = 1.85, *p* = 0.019, Receptive Language, β =  − 0.31, *SE* = 1.33, *p* = 0.005, and Expressive Language, β =  − 0.38, *SE* = 1.31, *p* < 0.001, such that higher toddler screen time was associated with lower developmental scores at 3–5 years. Screen time was also significantly associated with both autism and ADHD symptom scores: SRS total, β = 0.36, *SE* = 1.19, *p* = 0.002, and ADHD-RS total, β = 0.30, *SE* = 1.29, *p* = 0.009, such that higher toddler screen time was associated with more autism/ADHD symptoms at 3–5 years.

**Table 2:** Regression coefficients predicting outcomes at 3–5 years from screen time at 18 months longitudinally

Outcome	Estimate (β)	*SE*	*p*
MSEL Visual Reception T-score	−0.23	1.46	.044
MSEL Fine Motor T-score	−0.26	1.85	.019
MSEL Receptive Language T-score	−0.31	1.33	.005
MSEL Expressive Language T-score	−0.38	1.31	< .001
SRS Total Score	0.36	1.19	.002
Preschool ADHD Rating Scale Total Score	0.30	1.29	.009

*MSEL* Mullen Scales of Early Learning; *SE* standard error; *SRS* Social Responsiveness Scale

### Supplemental Analyses

To aid in the interpretability of analyses with standardized scores (i.e., MSEL subtests), we created dichotomous variables representing whether a child was in/above the average range (i.e., t-score ≥ 40) or below the average range (i.e., t-score < 40) and then conducted logistic regression analyses predicting dichotomous outcomes for each MSEL subtest from toddler screen time. There was no significant difference in the odds of falling below the average range at 3–5 years on MSEL Visual Reception (OR = 1.78, 95% Confidence Interval [CI; 0.92, 3.45]) or Fine Motor (OR = 1.60, 95% CI [0.96, 2.70]). There was a significantly increased likelihood of falling below the average range at 3–5 years on MSEL Receptive Language (OR = 1.91, 95% CI [1.12, 3.26]) and Expressive Language (OR = 2.19, 95% CI [1.23, 3.90]) with increased screen time.

## Discussion

We found evidence that autistic preschoolers and those with elevated symptoms of ADHD engaged in significantly more screen time earlier in development relative to those in a Comparison group of children who did not develop autism or early signs of ADHD. In addition, the Autism group and ADHD Concerns groups did not differ in terms of average screen exposure, revealing similarities across children with these neurodevelopmental challenges. On average, children later identified with autism or elevated symptoms of ADHD experienced more than double the amount of screen exposure at 18 months of age relative to the Comparison group. Similar to previously published findings [33], the majority of toddlers in our sample exceeded the AAP recommendation of “zero” before age 2, however, those with neurodevelopmental differences far exceeded the guideline and may be most vulnerable to experiencing early screen time. All children with Autism outcomes (100%) were exposed to screen time at 18 months, along with 94% of children in the ADHD Concerns group, and 74% in the Comparison group.

Because behaviors associated with autism and ADHD exist on a continuum and children who do not meet full diagnostic criteria may display some behaviors of either condition, particularly those with family histories of autism [43] or ADHD [6], it is important to evaluate not only group differences based on diagnostic thresholds, but to also consider symptoms continuously as they are distributed across the population. As such, we also took a dimensional approach to understanding associations between preschool symptoms and earlier screen time exposure, finding that across groups, more screen time at 18 months was associated with significantly greater autism and ADHD symptomatology at 3–5 years of age as reported by parents and significantly lower developmental scores based on a clinical assessment. Below average receptive and expressive language scores at age 3–5 were approximately twice as likely in children with higher amounts of screen time at 18 months.

Previous studies have found longitudinal associations in independent community samples between screen time and parent report of neurodevelopmental symptoms [1, 7, 18, 29, 58] and developmental achievement [32, 34, 56, 57, 65]. Our findings replicate and extend previous work, finding longitudinal associations between toddler screen time and preschool neurodevelopmental symptoms and developmental achievement in the same sample of children, including clinical outcome evaluation and direct assessment of children’s development.

It is important to note that we are unable to assess whether more screen time earlier in development contributes to increased neurodevelopmental symptoms or is secondary to other factors associated with developmental trajectories and behavioral phenotypes. Several factors could contribute to the associations between greater screen usage and atypical developmental outcomes documented in our sample. Research demonstrating associations between screen time and polygenic risk scores for autism and ADHD suggests that factors related to genetic phenotypes may play a role in screen media engagement [56, 57]. In addition, children with autism and ADHD may have greater difficulty regulating emotions [15] and behavior [3, 61], and therefore may be given access to greater screen time to help manage difficult behavior [46, 52]. Excessive screen time appears to negatively impact self-regulation, particularly in preschool aged children [9], and may exacerbate existing disruptive behaviors, further increasing the likelihood of more screen exposure. Moreover, autistic children show less interest in the social aspects of their environment [11, 28, 41], and both children with autism and ADHD have more difficulty regulating attentional engagement/disengagement [5, 50, 63]. These tendencies may disadvantage children from attending to cues that facilitate social interaction and may be exacerbated by screen time that reinforces inherent preferences for non-socially contingent audiovisual stimulation. In addition, toddlers with greater screen media exposure experience more disrupted sleep [25],reduced exposure to language [4, 8],and reduced interaction and play time with parents [27, 62], siblings [62], and peers [45] which could impact the acquisition of social, communicative, and self-regulatory skills, particularly in children who already experience increased challenges in these domains. This is especially important in the context of research indicating less optimal white matter development in areas of the brain that support language in preschoolers with greater screen exposure [23], less efficient acquisition of information presented via screen media in children under the age of 3 [54], and the relative benefit of live interaction in terms of word learning [53, 60].

Overall, young children with neurodevelopmental challenges may be exposed to greater amounts of screen time as the result of challenging behaviors and phenotypic preferences, and such increased exposure may have detrimental impacts on developmental achievement and symptom presentation regardless of clinical outcomes. However, comprehensive assessments in larger samples are required to fully characterize these associations. Our results highlight the importance of measuring clinical outcomes in future longitudinal studies of screen time and development to better characterize the associations and determine causality.

Our findings may have clinical implications for early intervention opportunities that could be incorporated into existing programs for children with developmental challenges. Although few randomized trials have examined screen time reduction interventions in relation to infant and toddler developmental outcomes, early findings are promising. For example, following a screen time reduction intervention in community samples, infants showed greater developmental gains [44] and preschoolers demonstrated improvements in aggressive behaviors [66] in comparison to those who did not receive the interventions. Case reports have documented marked autism symptom improvement following a reduction in screen time [16, 19], and several small, non-randomized intervention studies demonstrated reductions in repetitive behaviors [20, 26, 51], improvements in social communication skills [20, 26], and more typical EEG patterns [51] after providing parent training to reduce child screen time and increase parent–child engagement. Parents also report decreased stress following completion of a child screen time reduction intervention that included parent education and one-on-one support to increase parent–child social interactions [20]. Bolstering positive parent–child engagement, increasing parent efficacy to manage disruptive or challenging behaviors, and reducing parental stress are important considerations when asking parents to alter screen time practices within the home, given that parents may use screen time as a tool for managing child behavior and stress [46, 52]. Future research examining the efficacy of early screen time reduction interventions is needed.

Our results provide a window into screen time exposure in the context of developmental vulnerability, demonstrating that preschoolers with autism and those with elevated ADHD symptoms experienced more screen exposure prior to age 2 than children without these developmental challenges. The findings also provide additional evidence of longitudinal associations between early screen time and later neurodevelopmental symptoms and developmental functioning based on standardized clinical assessment, an aspect that has generally been lacking in prior studies, which has previously made associations difficult to interpret. To our knowledge, this is the first longitudinal study to examine screen time and development prospectively in toddlers with increased likelihood for atypical neurodevelopmental outcomes.

Our analyses were limited by our modest sample size and cannot establish the directionality of effects. Screen time was measured via parent report estimates and may not precisely capture the amount of daily screen time children received and instead may be more representative of overall tendencies. We did not capture maternal stress or depression, screen media content or quality (e.g., educational vs. entertainment), co-viewing habits, devices used, background television or parental screen time and were unable to examine relationships with these variables. We also did not measure screen time prior to 18 months of age and cannot establish whether children were more likely to receive higher screen time at 18 months if already exhibiting difficult behaviors, which could potentially relate to eventual diagnoses/outcomes.

Together with the existing literature, our findings underscore the importance of prioritizing future research to better understand both genetic and environmental influences on early development in children that may have compounding effects. Clarifying the direction of the association between increased screen time and increased symptoms is particularly important within the context of neurodevelopmental disabilities and may provide avenues for early intervention and prevention. Additional research in larger longitudinal samples using randomized designs including clinical outcome assessments will help clarify the direction of the associations between developmental outcomes and screen time patterns at various ages.

Our results may also further inform general parent education practices regarding screen time in very young children. Improved parental awareness of screen time guidelines and the potential negative impact of screen time on development in very young children is needed, including emphasis on the benefits of face-to-face interactions over video for learning in toddlers [53, 54, 60]. Parents report perceived educational benefits as a motivating factor for offering screen time [30, 49], and child-oriented programming is often marketed to parents. Conversely, parents who are aware of the AAP guidelines report less daily screen time for their children [30]. In addition to establishing guidelines and partnering with parents to devise plans to reduce the amount of time children view screen media early in life, offering parents behavioral tools to manage disruptive and/or challenging behavior, and strategies to replace screen time with social interaction and play, may benefit both children and parents. Randomized controlled trials assessing the effect of screen time reduction strategies on child development are needed to provide greater insight into associations between screen time and developmental outcomes in toddlers and preschool aged children.

## Summary

This study prospectively examined the daily amount of screen media exposure in 18-month-old children who later met diagnostic criteria for autism or demonstrated increased symptoms of ADHD at age 3–5 in comparison to children without these outcomes. We also examined associations between 18-month screen time and neurodevelopmental symptoms and developmental achievement at age 3–5 across the sample as a whole. Our findings support previous research demonstrating an association between greater screen time, diminished developmental achievement and increased neurodevelopmental symptoms in preschool aged community samples and extends the current body of knowledge by analyzing clinically determined neurodevelopmental outcomes and developmental achievement in relation to screen time within the same sample using a longitudinal study design. In our study, 18-month-olds with later identified neurodevelopmental differences were significantly more likely to experience screen time in greater magnitude compared to children who did not exhibit neurodevelopmental challenges later in development. In addition, across the sample, 18-month screen time was associated with higher scores on measures of neurodevelopmental symptoms and lower developmental achievement at age 3–5. Future studies designed to examine the direction of the associations between early screen time and developmental outcomes in children with and without neurodevelopmental challenges are needed, as well as research investigating the impact of early screen time reduction interventions on later developmental achievement.

## Data Availability

No datasets were generated or analysed during the current study.

## Footnotes

1 Due to small cell sizes within individual race categories, we defined of race as White vs. Non-white but recognize the limitations to this approach. Racial distribution: *n* = 1 American Indian or Alaskan Native, *n* = 3 Asian, *n* = 0 Black or African American, *n* = 20 more than one race, *n* = 0 Native Hawaiian or Other Pacific Islander, and *n* = 4 Unknown/Not Reported.
